# Shenfu injection alleviates the clinical symptoms of heart failure patients combined with conventional treatment: A protocol for systematic review and meta-analysis of randomized clinical trials

**DOI:** 10.1097/MD.0000000000023736

**Published:** 2021-04-16

**Authors:** Ziqing Luo, Min Jiang, Su Liu, Yunbiao Duan, Jianhui Huang, Huizhen Zeng

**Affiliations:** a The Laboratory Animal Centre of Guangzhou University of Chinese Medicine; b The Second Clinical College of Guangzhou University of Chinese Medicine; c Artemisinin Research Center, Guangzhou University of Chinese Medicine, Guangzhou; d Affiliated Jiangmen Traditional Chinese Medicine Hospital of Ji’nan University, Jiangmen; e Shenzhen Traditional Chinese Medicine Hospital, Shenzhen, Guangdong, China.

**Keywords:** heart failure, meta-analysis, protocol, Shenfu Injection, systematic review

## Abstract

**Background:**

Shenfu Injection (SFI) is a promising alternative treatment for heart failure (HF) in China. Many complete clinical trials have examined the efficacy of SFI combined use with conventional treatment (CT) vs CT alone. This study is to conduct a systematic review and meta-analysis of randomized clinical trials to evaluate the benefits and risks of using SFI in addition to CT for the treatment of HF.

**Method:**

All the trials will be searched through 4 English databases (MEDLINE via PubMed, the Cochrane Library, EMBASE, Web of Science) and 4 Chinese databases (the China Science and Technology Journal Database, Chinese Biomedical Literature Database, Wan-fang Database, the China National Knowledge Infrastructure.) from October 2005 to June 2019. Conference articles or articles with incomplete data will be removed. The primary outcome was the New York Heart Association, 6-minute walk test, hospitalization or rehospitalization. Left ventricular ejection fraction percentage, left ventricular end-diastolic dimension, Cardiac index, heart rate, N-terminal pronatriuretic peptide and other indicators were also assessed. RevMan V.5.3 Software and Stata 13.0 Statistics Software were used to calculate the data synthesis and conduct meta-analysis according to the guideline of Preferred Reporting Items for Systematic Reviews and Meta-analyses Protocols 2015.

**Results:**

Mortality, New York Heart Association function classification, heart rate, 6-minute walk test, hospitalization or rehospitalization, Heart rate, systolic blood pressure, DBP, Cardiac minute volume, left ventricular ejection fractions percentage, left ventricular end-diastolic dimension (mm), N-terminal pronatriuretic peptide, etc. and adverse effects will be comprehensively assessed to evaluate the adjunctive effect of SFI through different aspects. We will perform a meta-analysis of each outcome with subgroup analysis based on the type of HF, treatment methods, and course of disease. Sensitivity analysis will be conducted with clinical factors, treatment methods, methodological characteristics, and statistical heterogeneity (if applicable).

**Conclusion:**

This study will assess the adjunctive effect of SFI and its safety on HF with clinical evidence.

**PROSPERO registration number:**

PROSPERO CRD42020151856

## 1 Introduction

Heart failure is a well-recognized public health problem urging for significant and effective solutions. Poor clinical symptoms, low quality of survival life, high rate of hospitalization and unbearable complications of pharmacological management lead to huge burden for physicians and healthcare systems.^[1,2]^ Despite recent advances on heart failure (HF) treatment, its incidence still remains up to 2.2% in US, and By 2030, the prevalence in US is expected to increase to 2.97%.^[3]^ Thus, effective management and advanced treatment are proposed each year by American Heart Association in order to improve the poor symptoms or deterioration with HF patients.

Although the step-wise introduction of a variety of pharmacological treatments are recommended and evidence based, clinical symptoms of many HF patients still aggravate gradually with treatment, especially congestive heart failure patients with reduced ejection fraction remains a worse outcome.^[4,5]^ Furthermore, with the prolonged medication time, some adverse effects seriously affect the quality of survival life and the progress of the disease.^[6]^ Thus, many alternative and newly-developed drugs for HF have been widely applied in clinic, but it still lacks comprehensively and systematically evaluation based on clinical evidence for its efficacy and safety.

Chinese patent medicine served as an alternative therapy has been applied in clinic for a long time in Chinese healthcare systems. It was widely accepted by patients and physicians now because of its enhancement effect on alleviating clinical unbearable symptoms in effect. More than 650 clinical trials had demonstrated Chinese patent medicine Shenfu injection (SFI) has a significant effect on improving the symptoms of HF patients and alleviating the outcome of hospitalization.^[7–9]^ SFI combined with conventional treatment showed better effects on clinical efficacy, mortality, heart rate, N-terminal pronatriuretic peptide and 6-minute walking distance. Echocardiography results also showed that SFI combined with conventional treatment can improve cardiac function in patients with HF.^[10–13]^ Clinical research on SFI for HF are still conducted up to now, but their findings have been inconsistent, and lack comprehensive approach to assess its efficacy and safety successfully based on such a large number of SFI clinical studies data.

Thus, based on critical evaluation, we conducted a systematic review and meta-analysis of the available evidence to provide instructions on its efficacy and safety for the clinical application of SFI. In our research, we focus on the following questions:

(1) Is the combination of SFI and conventional treatment associated with enhanced effect in improving clinical symptoms or other indications including hospitalization rate and mortality rate of HF?
(2) How does SFI play its role in this process in terms of clinical indications?
(3) What kinds of HF patients are the best for SFI?

## 2 Methods

### 2.1 Inclusion and exclusion criteria for participants

Both male and woman patients with HF irrespective of any type (acute or chronic, left or right) were included, their ages were 18 to 85, and they were diagnosed with the American College of Cardiology Foundation/American Heart Association Guideline for the Management of Heart Failure or the other version of diagnostic criteria. All gender or race will be included but not pregnant patients. Besides, patients with cardiac implant device therapy were also excluded.

### 2.2 Interventions and comparison

In eligible researches, the experiment group was treated with SFI alone or combined with conventional western medicine (all guideline-recommended drugs: Angiotensin-converting enzyme inhibitors, β- blocker, angiotensin II receptor blockers, etc., administered alone or in combination), There were no restrictions on frequency and duration at first, because we will conduct subgroup analysis according them if they had a high heterogeneity. The comparison treatment in control group could be conventional medication alone or even placebo treatment.

### 2.3 Primary and secondary outcomes measures

The primary outcome was mortality, New York Heart Association function classification, heart rate, 6-minute walk test, hospitalization or rehospitalization. Besides, left ventricular ejection fraction, left ventricular end-diastolic dimension, Cardiac index, heart rate, systolic blood pressure, DBP, BNP, cardiac minute volume, NT-BNP, and other indicators were also assessed as secondary results. We will also conclude the number and proportion of adverse events in both groups to assess the safety of SFI application in clinic.

### 2.4 Included study design

Random control trials (RCTs) are considered as the best trails to provide evidence-based clinical data with unbiased information. Thus, we will only include RCTs, which would provide simple or specific stochastic method. Quasirandomised RCTs and non-randomised studies (eg. case–control studies, cohort studies, case studies etc.) were excluded. There is no restriction imposed on language, sample size, or publication status.

### 2.5 Search strategy and study selection

**Figure 1:**
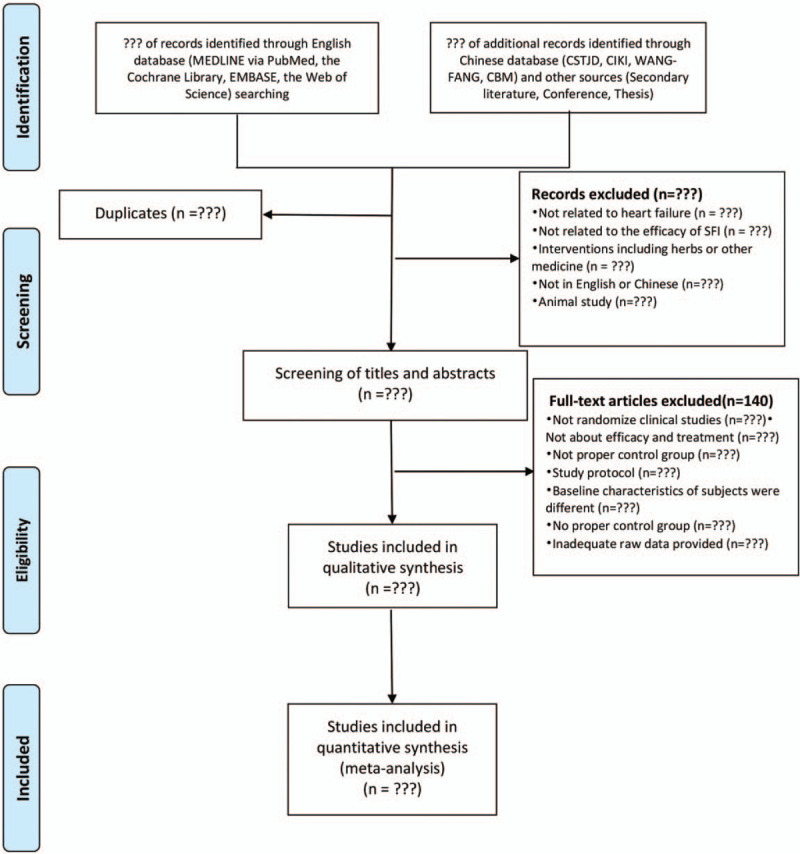
PRISMA flow chart of study selection process in the systematic review.

Two independent reviewers (LZQ and LS) search the published literature with search topics (P + I + C +O + S, P + I + C + O, and P + I + O), which we hope we could search for literature related to SFI on HF treatment as comprehensively as possible via 4 English databases (MEDLINE via PubMed, the Cochrane Library, EMBASE, the Web of Science) and 4 Chinese databases (the China Science and Technology Journal Database, Chinese Biomedical Literature Database, Wan-fang Database, the China National Knowledge Infrastructure.) from October 2005 to June 2019. The other 2 reviewers (JM and DYB) will screen all hits independently according to the titles and abstracts. Full texts of potentially eligible articles will be downloaded for full-text review and further evaluation. Studies with incomplete or insufficient detail in results or methods will be excluded, any exclusion of the article would be explained in the systematic review and meta-analysis flow diagram (as Fig. 1). Any disagreement will be resolved by discussion and consensus among reviewers.

### 2.6 Data extraction and quantitative synthesis

Two independent reviewers (HJH and ZHZ) conduct all the data extraction and extract patient characteristics according to the predesigned forms (as Table 1). In the extracted table, detail information about published year, author name, sample size, course of disease, age, method of treatment, duration, outcomes or indicators, and adverse events would be recorded. A third reviewer will access the implementation process. The SD_change_ and Mean_change_ would be calculated according to the following formulation if necessary. Besides, if the trials contained 3 or more time points for indicator value report, only 2 of them would be chosen for analysis, 1 of which is the baseline time. *I*^*2*^ will be used to assess heterogeneity to choose the effects model, *I*^*2*^ < 50 with fixed-effects model and *I*^*2*^≥ 50 for random-effects model.

$$Mean_{Change}=Mean_{End\;time}-Mean_{Baseline}$$

$$SD_{Change}=\sqrt{(SD_{baseline}^{2}+SD_{End\;time}^{2})-2\times R_{ratio}\times SD_{baseline}\times SD_{End\;time}}$$

**Table 1:** Characteristics of included RCTs investigating the effect of SFI on heart failure.

				Interventions				
Include study (author/year/language)	Sample(E/C)	Average age (E/C)	Duration	experiment group	control group	Diagnostic criteria	Adverse effects	Outcome Indicators
Example:								
Mosterd A, etc. 2010	60/60	48.56±12.78/48.97±13.33	7.53±5.78	SFI+CT	CT	2013 AHA	None	IL-6, IL-8, CRP, NT-proBNP, SBP (mmHg),DBP (mmHg),HR (bite/min), LVEF (%),SV (ml),CO (l/min),
2								
3								
4								
………								

### 2.7 Risk of bias assessment

We use the Cochrane Risk of Bias tool to evaluate bias of the included studies by 2 independent reviewers (LZQ, JM). The Cochrane Risk of Bias items include random sequence generation and allocation concealment [selection bias]; binding of participants and personnel [performance bias]; binding of outcome assessment [detection bias]; incomplete outcome data [attrition bias]; selective reporting [reporting bias]; and other potential sources of bias. Methodological quality was also assessed by RevMan software (Version 5.3).

### 2.8 Heterogeneity assessment and subgroup analysis

The chi-squared test will be applied to evaluate the heterogeneity. If *I*^*2*^>50%, trials would be considered with significant heterogeneity, and subgroup analysis will be necessarily performed to assess the potential heterogeneity sources, these sources possibly include the onset time, duration, type of HF, etc.

### 2.9 Sensitivity analysis and test sequential experiment analysis

In order to illustrate credibility of our results, sensitivity analysis and test sequential experiment analysis will be conducted. which aims to rule out the possibility of false positives and evaluate the robustness and reliability of the combined results of included studies.

### 2.10 Assessment of reporting biases

We will conduct analysis of Egger publication bias plot and Begg funnel plot with pseudo 95% confidence limits to determine the publication bias in all the literature with sufficient studies.

### 2.11 Ethics approval

All the data will be extracted from the published studies through database without directly relate to patients’ data, thus not ethical approval is required.

## 3 Discussion

Heart failure has been a worldwide cardiovascular disease, causing high burden for medical organization and personnel for its treatment. Although the current medical treatment can improve the survival rate of patients with HF, the rehospitalization rate and absolute mortality rate remain high with 5-year survival rate up to 50%.^[14]^ Heart failure has a long course, and the condition is easy recurrent, which seriously reduces the patient's quality of daily life and even threatens the patient's life, besides, mortality of HF patients with reduced ejection fraction (HFrEF) after discharge also increased from 4.3% to 6.4%.^[15]^

Exploration on the therapeutic effect of drugs from the etiology and pathogenesis of HF has always been a difficulty in its research field. There have been increasing trends and reports of Chinese patent medicine in the treatment of HF in the past 10 years, which showed a relatedly better efficacy on HF treatment.^[16,17]^ Until now, it has been found that there are more than 20 kinds of Chinese patent medicines that have a pharmacological effect on heart failure and improve the clinical symptoms of patients. Application of Qi Li Qiang Xin capsule on the treatment of HF was even rated as the 2013 annual highlight by the authoritative cardiovascular magazine “Journal of the American College of Cardiology.”^[18]^ Chinese patent medicine has become a newly kind of treatment on heart failure.

In recent years, there have been clinical reports that SFI have a good pharmacological effect in heart failure, its clinical application effect is accurate without serious adverse effects, which has aroused interest in the clinical research of SFI. However, the quality of the clinical efficacy research of SFI medicines is relatively inconsistent, and its research methods lack a unified standard. More clinical trials based on evidence-based medicine are still needed to be scientifically evaluated their efficacy and safety. Our study may provide a high-quality clinical evidence to demonstrate the effect of SFI on improving the clinical symptoms and reducing the occurrence of adverse events of HF patients. In addition, whether SFI combined with conventional treatment can be more effectively to improve the primary and secondary outcomes will be verified.

## Author contributions

Yunbia Duan designed the study and serve as an arbiter for a final decision throughout the entire procedure. Ziqing Luo and Su Liu perform data search, extraction and writing. Su Liu, Yunbiao Duan and Jianhui Huang performed data analysis and evaluated the accuracy of the whole process. Ziqing Luo, Min Jiang and Su Liu provided support for modifications of English writing. All authors contributed to the evaluation of review and analysis and approved the final version submitted for publication.

**Conceptualization:** Yunbiao Duan.

**Data curation:** Ziqing Luo, Min Jiang, Su Liu, Huizhen Zeng.

**Formal analysis:** Min Jiang.

**Methodology:** Ziqing Luo, Min Jiang, Su Liu, Jianhui Huang.

**Project administration:** Yunbiao Duan.

**Visualization:** Yunbiao Duan.

**Writing – original draft:** Ziqing Luo.

**Writing – review & editing:** Yunbiao Duan.
